# Supplementary material for: Decreased Hippocampal Neurogenesis in Aged Male Wistar Rats Is Not Associated with Memory Acquisition in a Water Maze
Source: Int J Mol Sci. 2023 Aug 26;24(17):13276. doi: 10.3390/ijms241713276 (PMC10487931; doi:10.3390/ijms241713276)
Supplement: Supplementary file 1 [file ijms-24-13276-s001.zip › ijms-2562304-supplementary.pdf]

**Table S1.** Behavior of 4 and 24-month-old rats in the open field tests 1 and 2

| Group | 4-month | | 24-month | |
|---|---|---|---|---|
| **Indices** | **OFT1** | **OFT2** | **OFT1** | **OFT2** |
| Crossings | 83.2 ± 1.5 | 45.4 ± 10.4 [aaa1] | 42.2 ± 4.8 [bbb] | 26.5 ± 5.5 [a] |
| Rearing | 21 ± 1.5 | 8 ± 1.1 [aaa] | 19.0 ± 3.8 | 12.2 ± 2.8 [a] |
| Latency | 7.4 ± 1.1 | 14.9 ± 6.7 | 5.7 ± 0.8 | 3.2 ± 0.4 |
| Entries to center | 1.2 ± 0.3 | 0.7 ± 0.2 | 1.1 ± 0.5 | 0.4 ± 0.2 |
| Grooming | 3.4 ± 0.4 | 3.0 ± 0.5 | 2.0 ± 0.4 | 2.9 ± 1.0 |
| Defecation boli | 2.4 ± 0.6 | 3.2 ± 0.5 | 1.7 ± 0.6 | 2.3 ± 0.6 |

[1] Differences are significant at [a] – p < 0.05 and [aaa] – p < 0.001 OFT1 vs. OFT2 or [bbb] – p < 0.001 4-m vs. 24-m according to post hoc Tukey HST test.

**Table S2.** Data on RM ANOVA for behavioral indices in the open field test

| Factors / Indices | Age | OFT1-2 | Age×OFT1-2 interaction |
|---|---|---|---|
| Crossings | F(1,29) = 21.3; p = 0.00007 | F(1,29) = 61.1; p = 0.0000 | F(1,29) = 9.02; p = 0.005 |
| Rearing | F(1,29) = 0.10; p = 0.75 | F(1,29) = 47.2; p = 0.0000 | F(1,29) = 4.24; p = 0.048 |
| Latency | F(1,29) = 1.77; p = 0.19 | F(1,29) = 0.34; p = 0.56 | F(1,29) = 1.40; p = 0.25 |
| Entries to center | F(1,29) = 0.63; p = 0.43 | F(1,29) = 5.001; p = 0.0332 | F(1,29) = 0.096; p = 0.758 |
| Grooming | F(1,29) = 0.93; p = 0.34 | F(1,29) = 0.305; p = 0.58 | F(1,29) = 2.68; p = 0.11 |
| Defecation boli | F(1,29) = 1.096; p = 0.303 | F(1,29) = 1.76; p = 0.195 | F(1,29) = 0.44; p = 0.84 |
